# Microwave Resonant and Zero-Field Absorption Study of Doped Magnetite Prepared by a Co-Precipitation Method

**DOI:** 10.3390/molecules19068387

**Published:** 2014-06-19

**Authors:** Juan Carlos Aphesteguy, Silvia E. Jacobo, Luis Lezama, Galina V. Kurlyandskaya, Nina N. Schegoleva

**Affiliations:** 1 LAFMACEL, Fac. de Ingeniería, UBA, Paseo Colón 850, C1063EHA Buenos Aires, Argentina; E-Mails: caphestegu@fi.uba.ar (J.C.A.); silviajacobo@gmail.com (S.E.J.); 2 Depto. Química Inorgánica, Universidad del País Vasco UPV-EHU, 48940 Leioa, Spain; E-Mail: luis.lezama@ehu.es; 3 Depto. de Electricidad y Electronica, Universidad del País Vasco UPV-EHU, 48940 Leioa, Spain; 4 Dept. of Magnetism and Magnetic Nanomaterials, Ural Federal University, 620000 Ekaterinburg, Russia; 5 Institute of Metal Physics UD RAS, 620041 Ekaterinburg, Russia

**Keywords:** magnetic nanoparticles, co-precipitation method, magnetite, magnetic properties, microwave absorption

## Abstract

Fe_3_O_4_ and Zn_x_Fe_3−x_O_4_ pure and doped magnetite magnetic nanoparticles (NPs) were prepared in aqueous solution (Series A) or in a water-ethyl alcohol mixture (Series B) by the co-precipitation method. Only one ferromagnetic resonance line was observed in all cases under consideration indicating that the materials are magnetically uniform. The shortfall in the resonance fields from 3.27 kOe (for the frequency of 9.5 GHz) expected for spheres can be understood taking into account the dipolar forces, magnetoelasticity, or magnetocrystalline anisotropy. All samples show non-zero low field absorption. For Series A samples the grain size decreases with an increase of the Zn content. In this case zero field absorption does not correlate with the changes of the grain size. For Series B samples the grain size and zero field absorption behavior correlate with each other. The highest zero-field absorption corresponded to 0.2 zinc concentration in both A and B series. High zero-field absorption of Fe_3_O_4_ ferrite magnetic NPs can be interesting for biomedical applications.

## 1. Introduction

In recent years the study and development of nanosized magnetic nanoparticles (NPs) has received growing attention both for understanding their fundamental properties and for their technological, environmental, and biomedical applications [1,2,3,4,5]. All these applications require strict control over the composition, morphology of particles and the reduction of their dimensions is an important parameter defining the structural properties and the dynamical behavior of the NPs. One growing direction of the research is to study how the magnetic properties of NPs are modified with the change of their shape and composition. The differences in a magnetic behavior are expected to arise from the contribution of surface properties [1] and interparticle interactions among other factors. The last depends on the non-magnetic matrix in which NPs are suspended, and on the size confinement effects, essentially due to the surface anisotropy of the particles [4,6]. Magnetic NPs with different and controlled sizes and properties can be prepared by various chemical and electrophysical methods like sol-gel, co-precipitation, laser evaporation, electric explosion of wire, *etc.* [1,4,5,6,7,8,9].

Ferrimagnetic iron oxides (Fe_2_O_3_ and Fe_3_O_4_) and zinc ferrite (Zn_x_Fe_3−x_O_4_) are among the most studied NPs systems because of their excellent magnetic and electric properties [6,7,8,9,10,11]. The first mentioned has unique chemical stability, biocompatibility, low cost and good magnetic response. Furthermore, Fe_2_O_3_ has a cubic inverse spinel structure where two thirds of the iron ions are Fe^3+^: half of them occupy A sites (tetrahedral sites) and the other half occupy B sites (octahedral sites). The remaining Fe ions are Fe^2+^ and occupy B sites. Magnetite has an ordering temperature of T_N_ = 860 K and a room temperature (RT) saturation magnetization *M*_s_ = 92 emu/g for the bulk state [12,13,14,15]. Doping magnetite with transition metal elements allows the modification of quantities such as conductivity and saturation magnetization [9,13].

There have been many attempts to employ microwave techniques to study NPs [16,17,18,19]. Ferromagnetic resonance (FMR) is one of the basic tools to study the magnetic properties of ferromagnets. It is capable of yielding information concerning crystalline anisotropy, effective magnetization, stresses amd magnetic inhomogeneities. In a ferromagnetic material, the electrons participating in the resonance process are the 3d- shell electrons which are responsible for the ferromagnetism. In a typical experimental arrangement the ferromagnetic specimen in the shape of a thin sheet or foil is employed as one wall of a rectangular cavity terminating a wave guide fed by a microwave generator. The ferromagnetic side of the cavity is chosen so that the magnetic vector of the microwave field is constant in direction in the plane of the wall. A static magnetic field is applied in the plane of the wall but perpendicular to the microwave magnetic field [1,12,19,20,21]. Ordinarily the ferromagnetic resonance of a spherical sample is calculated under the assumption that the oscillating part of the magnetization is the same at all points of the sample. Multiple ferromagnetic resonances have been observed [16] under conditions when higher special modes were excited.

Absorption of radiation in a ferromagnet is governed by the field dependence of the dynamic permeability μ. The microwave skin depth *δ* ~ (2*ρ*/*μω*)^1/2^, where ρ is the resistivity and *ω* is the angular frequency of the electromagnetic radiation [9,18]. Thus, the resistivity plays an important role. In the case of ferrites for microwave devices, the line width of the ferromagnetic resonance, Δ*H*, related to the energy losses in the material is a very important parameter to be taken into account. FMR studies of single crystals of magnetite Fe_3_O_4_ give a solid basis for FMR studies of iron oxide NPs [19].

Nanoparticles functionalized by chemotherapy drugs play the role of carriers for delivering the drugs to tumor cells with minimum damage to healthy cells. They also can be employed in hyperthermia and thermal ablation—promising forms of cancer therapy [22]. These treatments employ the physical phenomenon called enhanced permeability and retention, *i.e.*, the tendency for MNPs to accumulate selectively in tumor tissue without damaging healthy cells [23]. Recently new approaches related to the effectiveness of the cancer drugs were introduced. One of them is the possibility to take advantage of previously unexploited weaknesses in tumor architecture in order to increase the effectiveness of the nanodrugs. Blood vessel structure in malignant tissue differs from the structure of healthy vessels. The blood vessels in tumors have irregular and larger gaps. Generally, the gap size in tumors ranges from a few hundred nanometers to a few microns [24]. This means that nanoparticles of 10 to 300 nm in diameter can be appropriate for cancer therapy. This approach renewed interest in biocompatible MNPs with bigger size and their methods of fabrication.

Another request of nanomedicine related to MNPs is thorough characterization of size, shape, surface features, state of aggregation or agglomeration and magnetic properties [1,23]. The same parameter is expected to be measured with multiple methods to gain a detailed understanding. The microwave technique is an important addition to the traditionally employed methods for MNP characterization. Electrical transport, magnetic properties and FMR studies of magnetite and zinc ferrite nanostructures have attracted special interest for the development of bio- and technological materials with tunable physical properties [25,26].

In this work we have fabricated two series of Fe_3_O_4_ and Zn_x_Fe_3−x_O_4_ NPs with different compositions. They were prepared by a co-precipitation method under different conditions. Their structural, magnetic and microwave properties were studied as a function of Zn concentration.

## 2. Results and Discussion

### 2.1. XRD and TEM Characterization

**Figure 1:**
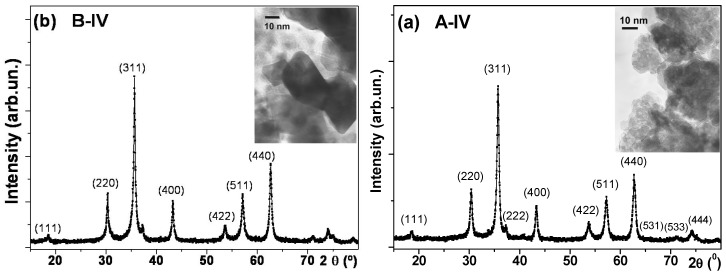
X-Ray diffraction spectra and TEM images for sample A-IV (Zn_0.5_Fe_2.5_O_4_) prepared in water (**a**) and sample B-IV (Zn_0.5_Fe_2.5_O_4_) prepared in water with ethanol (**b**).

Figure 1a,b shows the X-ray diffraction spectra and TEM micrographs of Zn_0.5_Fe_2.5_O_4_samples belonging to series A and B. All measured NPs have very similar XRD profiles. We observed that only Bragg reflections (in brackets) belonging to the cubic spinel phase appeared (Fd3m), indicating that the samples were single phase materials (JCPDS 19-629). The information from broadened X-ray diffraction lines is normally used to estimate the average size of coherent diffraction domains by using the Scherrer approach. In all cases, the deconvolution of not overlapped diffraction maxima (at 30.3° and 33.3°) in 2*θ* provides the half-width parameter for each XRD diagram (Table 1).

**Table 1:** Description and selected properties of the samples: series A-preparation in aqueous solution; series B-preparation in water-ethyl alcohol mix; *D*_XRD_: average grain size determined by X-ray data analysis, *M*_s_-saturation magnetization.

Sample	Composition	*D*_XRD_ (nm)	*M*_s_ (kOe)
A-I	Fe_3_O_4_	~60	4.32
A-I-talc	Fe_3_O_4_ (1:3 parts of talc)		
A-II	Zn_0.1_Fe_2.9_O_4_	~57	4.19
A-III	Zn_0.2_Fe_2.8_O_4_	~43	5.46
A-IV	Zn_0.5_Fe_2.5_O_4_	~22	3.25
A-IV-talc	Zn_0.5_Fe_2,5_O_4_ (1:3 parts of talc)		
B-I	Fe_3_O_4_	~42	3.49
B-II	Zn_0.1_Fe_2.9_O_4_	~37	4.20
B-III	Zn_0.2_Fe_2.8_O_4_	~58	4.49
B-IV	Zn_0.5_Fe_2.5_O_4_	~38	4.49

**Figure 2:**
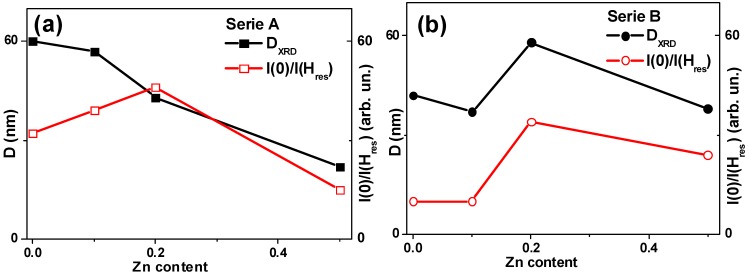
Crystallite size *D*_XRD_ obtained from the XRD analysis and evolution of zero field absorption as a function of Zn concentration for series A (**a**) and B (**b**) samples.

Series A: as Zn substitution (x) increases, the mean crystallite calliper size *D* progressively decreases from 60(4) nm to 22(2) nm (Figure 2a). For series B the observed behaviour is more complex. First of all, size *D* depends non-monotonously on the zinc quantity and the synthesis seems to be less stable: the x decrease results in the *D* change in the range between 42(3) nm for x = 0.0 and 38(3) nm for x = 0.5 (Figure 2b).

TEM studies of all samples also confirmed phase uniformity, namely, the fact that the crystalline structure is a cubic (Fd3m) phase. The average *D* sizes for B series were smaller compared with average sizes of A series in accordance with XRD studies. Insets of Figure 1 show typical TEM data for selected samples. TEM images let us conclude that the particles obtained in water tend to have cubic shape, while those obtained in water-ethanol look more spherical-like. In all A series cases it was observed that the inclusion of Zn causes a decrease in the size of the crystal being in good agreement with XRD determinations. In B series the size distribution was larger, what made TEM statistics less representative.

### 2.2. SEM

**Figure 3:**
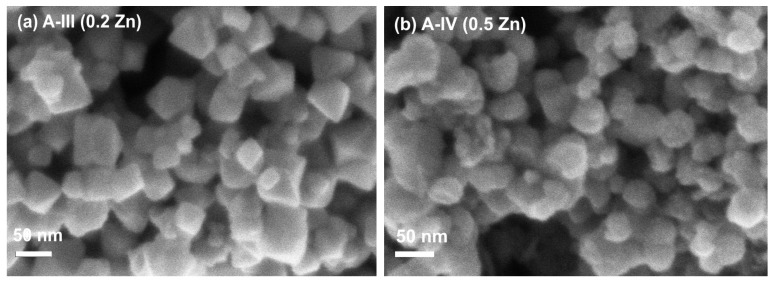
SEM images of sample A-III (Zn_0.2_Fe_2.8_O_4_) (**a**) and sample A-IV(Zn_0.5_Fe_2.5_O_4_) (**b**).

Figure 3 shows two examples of the morphology of Zn_0.2_Fe_2.8_O_4_ and Zn_0.5_Fe_2.5_O_4_ samples of A type studied by SEM. One can appreciate the cubic shape in both cases in good agreement with TEM data. Again, the average crystallite size decreases when the amount of Zn increases in a good agreement with XRD analysis.

### 2.3. Magnetic Properties

**Figure 4:**
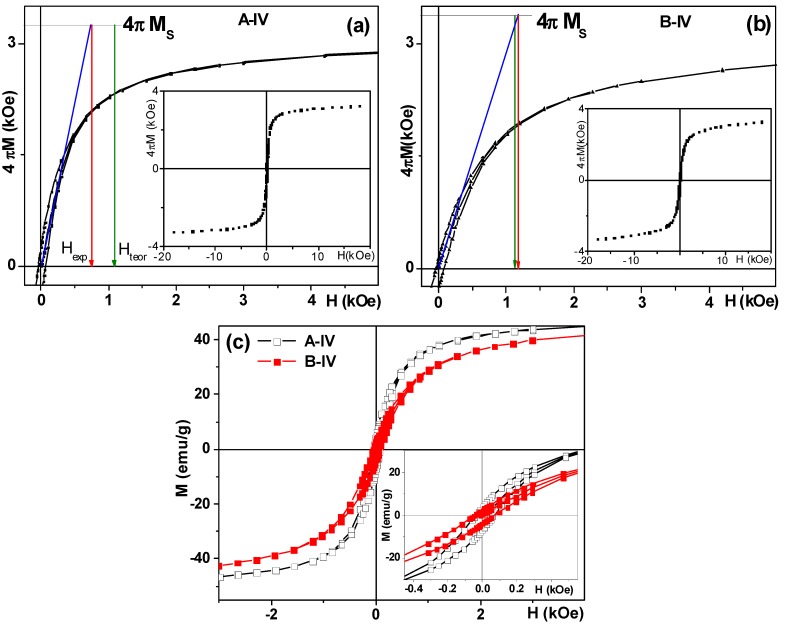
Hysteresis loops and primary magnetization curves of A-IV (**a**) and B-IV (**b**) samples. Insets for (**a**) and (**b**) show hysteresis loops of the same nanoparticles measured at room temperature up to higher field. Hysteresis loops (magnetization recalculated for emu/g) for A-IV and B-IV in small field clearly indicating a non-zero coercivity (**c**).

Figure 4 shows as an example parts of the hysteresis loops corresponding to positive magnetic fields with primary magnetization curves of the samples A-IV and B-IV indicating that we are dealing with soft ferrites (the saturation fields were close to 10 kOe). The inclusion of Zn modified magnetic parameters. Saturation magnetization increases up to x = 0.2 (see Table 2).

Let us analyze the saturation magnetization, *M*_s_, values. It has been shown in many previous studies that *M*_s_ of the iron oxide NPs can be significantly smaller compared to corresponding bulk values showing a clear tendency: the smaller the particles, the lower the value of the saturation magnetization [1,7,9]. The widely accepted explanation of a decrease in saturation magnetization as the average particle size reduces is due to the existence of surface spin disorder [1,9] and a nonsufficient number of nearest neighbours seems to be valid for NPs of the studied types.

**Table 2:** Selected results of magnetic measurements: *M*_s_-saturation magnetization, *H*_C_-coercivity Δ*D*-deviation from the position for spherical nanoparticles.

Parameter	A-I	A-II	A-III	A-IV	B-I	B-II	B-III	B-IV
4π *M_s_* (kOe)	4.32	4.19	5.46	3.25	3.49	4.2	4.5	3.4
*H*_teor_ = *M*_s_/3	1.45	1.40	1.82	1.08	1.16	1.4	1.5	1.1
*H*_teor_/*H*_exp_	1.45/0.97	1.40/1.01	1.82/1.32	1.08/0.75	1.16/0.95	1.4/1.0	1.5/1.1	1.1/1.2
	1.495	1.39	1.38	1.44	1.22	1.5	1.4	1.0
Δ*D*	~50%	~40%	~38%	~45%	~22%	~46%	~37%	~5%

Let us assume a homogenous sample of equal volume non-interacting spheres of diameter (*D*) which, thus, has an equal surface to volume ratio, where *D* is the average crystal size for magnetite obtained from XRD data. Although complete saturation is not attained in the case of A and B series of NPs, the value of the saturation magnetization can be deduced using the saturation magnetization approach of the high field M(H) in order to insure the saturation magnetization value [1]. The initial slope of the 4π*M vs.* H curve (Figure 4a,b) gives possibility to evaluate the degree of deviation of the grains from sphericity.

For the grains of spherical shape the cut point of the straight line corresponding to extended primary magnetization curve and the straight line y = 4π*M*_s_ should appear at 4π*M* ≈ 3*H* [12]. Table 2 shows the summary of the analysis of the shape of primary magnetization curves. For example, for A-IV M 4π*M*_s_ ≈ 3.3 kOe (Table 2) and then *H*_teor_ = 1.1 kOe is a position for the spherical NPs instead we observe the cut point at *H*_exp_ = 0.8 kOe indicating the existence of deviations from sphericity.

The observed lack of saturation for the rather high field is also in accordance with the structural data. We detected the presence of very small NPs in TEM studies as well as a peculiar background in XRD spectra that give us the basis to suppose that very fine iron oxide MNPs are in a superparamagnetic state or have peculiar surface magnetic anisotropy (for example, a spin glass shell) [1]. At the same time it is absolutely clear that we are dealing with relatively big NPs of average size range between 22 and 60 nm. The magnetic behavior of ferro-/ferrimagnet can be subdivided on the basis of grain size into three ranges: multidomain, single domain and superparamagnetic state. It is commonly accepted that the grain size of about 30 nm is critical for a room temperature transition from single domain into superparamagnetic state for the appropriate times of the measurements [14]. Although in all cases superparamagnetic contribution is essential we also expect ferromagnetic contribution for all samples under consideration. Confirmation of this expectation comes from the shape of the room temperature hysteresis loops shoving linear approach to saturation but non-zero coercivity.

### 2.4. FMR

**Figure 5:**
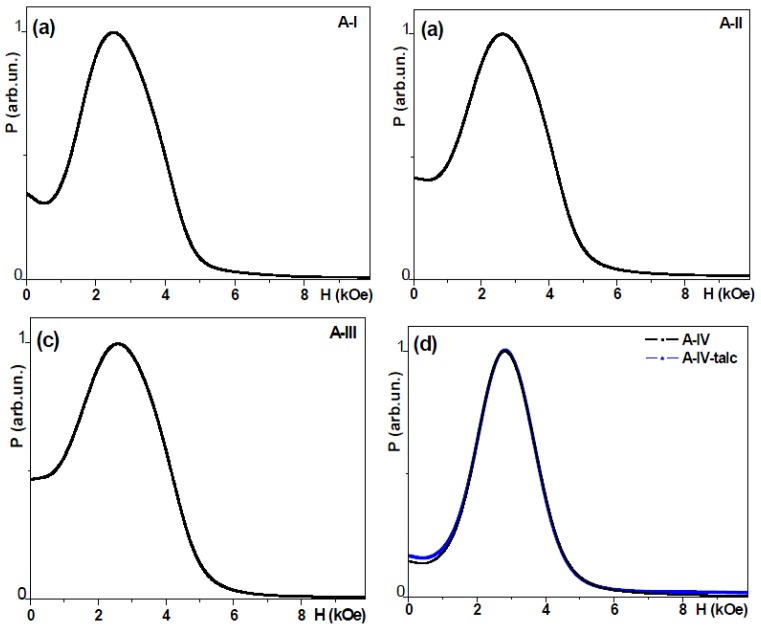
Microwave absorption as a function of the external magnetic field for Series A samples, *f* = 9.5 GHz.

**Figure 6:**
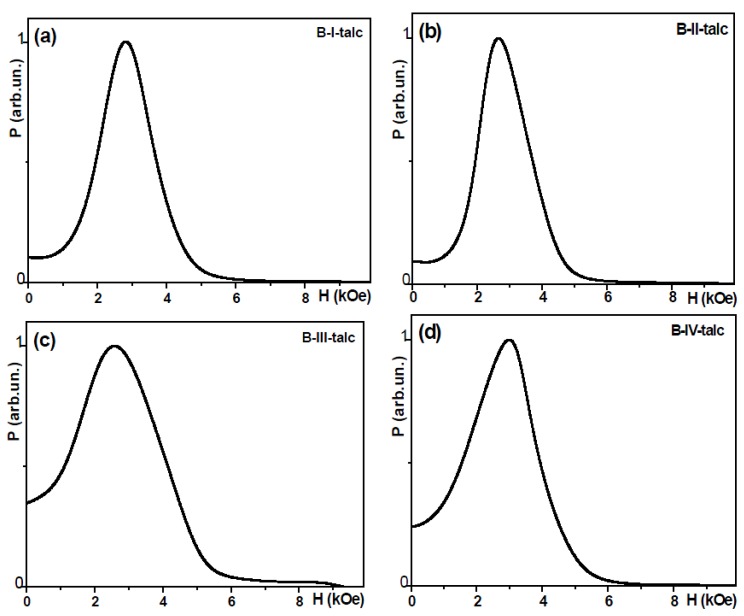
Microwave resonant absorption as a function of the external magnetic field for Series B samples, *f* = 9.5 GHz.

The location of samples for FMR studies was carefully controlled in order to insure zero contribution of microwave electric field. Resonance fields (*H*_res_) and linewidths (∆*H*) are listed in Table 3. FMR measurements confirmed in accordance with XRD and TEM studies, that NPs of both series are magnetically homogeneous materials: only one line was seen in every case (Figure 5 and Figure 6). At the same time the widths were quite large (∆*H* > 1 kOe). As before [9,10] it is obvious that the uncontrollable distributions of sizes, shapes and orientations are contributing into FMR line width. It is essentially an “envelope” of the resonances arising from individual grains. If the samples were spherical and the contributions from stresses were negligible, in the case of negligible magnetocrystalline anisotropy, the resonance field is given by the equation:

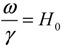
(1)
where *ω* = 2π*f* is the microwave frequency and γ is the gyromagnetic ratio where γ = 8.8 g (Mrad/Oe). For Fe_3_O_4_, *g* = 2.12 (*g—*spectroscopic Landé factor [20]) and *H*_0_ = *H*_res_ ≅ 3.27 kOe which is appropriate for magnetite, for *f* = 9.5 GHz [15]. The *H*_res_ values are known to be no better than 0.1 kOe and the Δ*H* values are good to about 10%.

**Table 3:** Microwave properties of A and B series samples with and without dilution (1:3 parts of talc); *f* = 9.5 GHz.

Sample	*H*_res_ (kOe ± 0.1)	∆*H* (kOe ± 0.2)	*I* (*H* = 0)/*I* (*H*_res_)
A-I	2.5	2.1	32
A-II	2.6	2.1	39
A-III	2.6	2.1	46
A-IV	2.8	1.8	15
A-IV-talc	2.8	1.8	16
B-I-talc	2.8	1.6	9
B-II-talc	2.6	1.6	8
B-III-talc	2.5	2.1	34
B-IV-talc	3.0	1.9	24

#### 2.4.1. Series A Samples

In the series A samples the value of the resonance field changes between 2.5 and 2.8 kOe for different samples and the half-power linewidth chages between 1.8 and 2.1 kOe. In all studied samples the resonance *H*_res_ lies below the one given by Equation (2): *H*_res_ = 3.27 kOe (for the frequency of 9.5 GHz) is expected for spheres. This difference indicats the presence of internal fields (*H*_int_) affecting the resonance field position and therefore one should write:

*H*_res_ = *H*_0_ + *H*_int_ (2)

The possible reason for the origin of *H*_int_ (0.2–0.5) kOe can be understood by invoking the same arguments as noted earlier: local dipolar fields, deviations from sphericity, magnetocrystalline anisotropy and stresses [4]. The presence of superparamagnetic phase (although not always in a noticeable way for such a broad lines) can also influence magnetostatic, FMR behaviour and low field microwave absorption. We previously estimated that two spherical grains of magnetite touching each other are creating a dipolar of the order between 0.4–0.6 kOe, which is sufficient to account for the higher concentration data in Table 2. For magnetite NPs several hundreds Oersted shifts were discussed previously even in the case of non-interacting magnetic NPs with cubic magnetocrystalline anisotropy and uniform space distribution of the easy axes.

To understand rather large linewidths one can take into account work of Kale *et al.* [19] in which they have found that even in single crystal Fe_3_O_4_ samples, the narrowest linewidths were of the order of 1 kOe, *i.e.*, rather high line widths is typical for ferrites.

#### 2.4.2. Series B Samples

In the B type samples the g value is not known, but it is not likely to be much different from 2.1 [9,26]. The average grain size decreases from B-I to B-IV is non-monotonus way with maximum *D* size corresponding to B-III sample (Figure 2). It is worth mentioning that in all cases of A and B type samples the average sizes of the NPs were of similar order (between 22 and 60 nm).

For the particles with significant shape deviation from a spherical one, even separation by mixing with talc (difference between Series A and Series B, see Figure 5 and Figure 6 and Table 3) does not result in the line displacement toward the “correct” position for Fe_3_O_4_: the resonance maximum is still displaced due to the shape anisotropy, the stresses [9] and possibly due to the contribution of the superparamagnetic phase as discussed above. Coming back to TEM and SEM micrographs one can see, that all samples contained a certain fraction of flattened NPs or NPs which are squared in shape (Figure 1 and Figure 3 ). The higher deviation of the *H*_res_ position from *H* = 3.27 kOe and higher width of the resonance lines for MNPs of A series are clearly justified by the shape anisotropy. NPs of B-IV type shows *H*_res_ closest to the position of spherical NPs (Table 3).

#### 2.4.3. Zero Field Absorption

In addition to the resonant response, all samples show significant zero field microwave absorption which requires high conductivity (Figure 5 and Figure 6) like it was previously observed in [19,27] or essential addition due to the contribution of the superparamagnetic part of the ensemble). The level of zero field absorption changes quite a lot for different samples. Although it affects the resonance position and resonance line width calculation, keeping in mind rather high line widths one can neglect this experimental error in the resonance position calculation. The origin and mechanism of the zero field microwave absorption was widely discussed earlier: the zero-field absorption arises from spins [1,9,21]. It is attributed to Joule heating in the grains being caused by the rapidly time varying radio frequency induction. This requires that the MNPs have a low resistivity which is rather unexpected for pure ferrite. At the same time in our previous work on NiCuZn ferrite obtained by autocombustion method [9] we did observe a rather high zero-field absorption and proposed a simple explanation based on the existence of non-uniform nanostructures (like Cu or Zn containing nanowires with enhanced conductivity) in ferrite grains. Another interesting example is given by Lorenz *et al*. [25] were they had shown a possibility to control conductivity at 300 K by the pulsed laser deposition conditions over 7 orders of magnitude giving the possibility to grow even semiconducting ZnFe_2_O_4_ spinels.

Especially high values of zero field absorption are typical for the samples of A series. In order to quantify zero-field absorption we have calculated the following parameter:

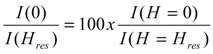
(3)
where *I*(*H* = 0) is the height of the resonance line in zero field and *I*(*H* = *H*_res_) is the height of the resonance line in the resonance field. Figure 2 shows the dependence of *I*(0)/*I*(*H*_res_) parameter on the concentration of the Zn, grain size data are also given for the sake of comparison.

For A type samples the grain size decreases with an increase of the Zn content. One could expect an increase of the zero field absorption with an increase of the concentration of zinc but the situation is much more complex. An important remark—we observe sizable zero-field absorption even in magnetite A-I sample which might be the consequence of non-stoichiometry and non-uniform iron distribution over the NPs. Again as in [9] we did not observe in TEM studies any structural evidence supporting this supposition, but microwaves unequivocally indicate the presence of elements with high conductivity. Incorporation of Zn results in the increase of the *I*(0)/*I*(*H*_res_) up to 0.2 zinc concentration which is logical due to high zinc conductivity. At the same time for A-IV sample with rather small grain size one can expect much more uniform distribution of both iron and zinc leading to a decrease of zero field absorption. High zero-field absorption of A-I ferrite can be useful for biomedical applications keeping in mind the biocompatibility of the pure iron oxides.

For B type samples the grain size and zero field absorption shows remarkable correlation. Again we observe sizable zero field absorption in the B-I magnetite sample. In accordance with previous discussion it is smaller in the sample B-I comparing with A-I probably because the average grain size is smaller in the case of B-I sample. It looks like that precisely bigger grains are the subject of non-uniform iron distribution over the NPs. As the average size of the grains is higher in B-IV case comparing with A-IV, the *I*(0)/*I*(*H*_res_) parameter is also higher for B-IV case. In both A and B series the highest zero-field absorption corresponded to 0.2 zinc concentration. More structural studies are necessary in order to understand the observed effect in details, but it is clear that the microwave technique is a powerful tool to reveal corresponding changes in the conductivity, perhaps related to the non-uniform structure of NPs.

## 3. Experimental

### 3.1. Samples Preparation

Two series of Fe_3_O_4_ and Zn_x_Fe_3−x_O_4_ nanoparticles were prepared by the co-precipitation technique [4]. Series A samples were prepared in aqueous solution and series B samples were prepared in water/ethyl alcohol mixtures (Table 3).

#### 3.1.1. Series A (A: I–IV) Samples

Several samples of pure magnetite and Zn-doped ferrite Zn_x_Fe_3–x_O_4_ were obtained by the co-precipitation technique following the procedure described in [9]. First of all two solutions (Sn_1_ and Sn_2_) were prepared in order to fabricate the magnetic NPs.

Sn_1_: Previously weighed amounts of KNO_3_ (oxidant agent) and KOH were completely dissolved in distilled water under nitrogen bubbling at room temperature for 15 min.

Sn_2_: Initially weighed amounts of FeSO_4_·7H_2_O and ZnSO_4_·7H_2_O salts were dissolved in distilled water and H_2_SO_4_ 2M concentration in front of acid under nitrogen bubbling at room temperature for 15 min.

As the next step Sn_2_ was slowly added to Sn_1_ with mechanical stirring and constant nitrogen bubbling (20 min more) at RT. Then the system sample-reactor was kept at rest inside a thermostatic bath at 70 °C during 210 min facilitating the formation of the spinel phase crystals. The sample was cooled at RT for 20 h (pH_final_ ≈ 9.0). The solid precipitates were separated by magnetic sedimentation, washed several times with distilled water, methanol, acetone and carefully centrifuged. Finally, the sample was dried in vacuum at 50 °C for 24 h. For Zn-doped samples FeSO_4_·7H_2_O was partially replaced by ZnSO_4_·7H_2_O in a fraction corresponding to the desired composition.

#### 3.1.2. Series B (B: I–IV) Samples

Magnetic NPs of B series were prepared following a similar procedure previously described for Series A samples. The difference in this case was that distilled water was replaced by a mixture of distilled water plus absolute ethanol.

#### 3.1.3. Sample Dilution

In some cases for magnetic and microwave studies, the NPs were intimately mixed with non-magnetic talc powder [1] in the mass ratio of 1 part in 3, in order to reduce agglomeration and interaction. Finally, the mixture, diluted with GE7031 varnish [4], was evenly spread onto a quartz plate (Table 1) as a “film” of a thickness of about 50 microns. Small disc (diameter of about 4 mm) was carefully cut from the “film” for microwave studies in the field applied in plane of the disc. It was previously shown [1,4,9] that such a way of preparation of samples causes the line narrowing in the case of essentially spherical NPs. Samples without dilution with talc (diluted with GE7031 varnish) were also measured for comparison. It is important to mention the importance of the filling factor, *i.e.*, how much of sample’s volume is occupied by the ferromagnetic material because in the case of considerable magnetic anisotropy (*i.e.*, shape anisotropy of the sample as whole) it can influence the slope of M(H) curves and shift the resonance lines. As before [1,4,9,12], we have prepared powder samples following well established protocol elaborated in previous studies of the FMR Group at the University of Maryland [1,21,27]: the filling factor for GE-NPs composite was estimated to be as high as 14% and for GE-NPs-talk composites as high as about 4%. Additional magnetic measurements for a magnetic field applied in plane of the disc and perpendicular to the plain of the disc showed that we can neglect the shape anisotropy of the sample as whole in this particular case.

### 3.2. Sample Characterization

For structural characterization the X-ray powder diffraction patterns (XRD) were collected using an X’PERT PRO automatic diffractometer (Philips, city, country) operating at 40 kV and 40 mA, in theta-theta configuration, secondary monochromator with Cu-K_α_ radiation (*λ* = 1.5418 Å) and a PIXcel solid state detector (active length in 2*θ* 3.347°). Data were collected from 15 to 80° 2*θ* (step size = 0.026 and time per step = 800 s, total time 2 h) at RT. A fixed divergence and antiscattering slit giving a constant volume of sample illumination were used.

The average crystallyte size of nanoparticles (Series B) was calculated for the strongest diffraction peak in the plane (311) from the line broadening using the Scherrer equation:

*β_hkl_* = *k ∙ λ/L_hkl_* ∙cos*θ* (4)

where *β*_hkl_ is the broadening of the diffraction line measured at half the line maximum intensity, *λ* is the X-ray wavelength, *L*_hkl_ is the crystal size, *θ* is the diffraction angle and *k* is the Scherrer shape factor (*k* = 0.9 was used for the calculations).

The morphology of all samples was examined by scanning electron microscope (SEM, DSM 982 GEMINI, Zeiss, Oberkochen, Germany) and transmission electron microscopy (TEM) was performed after the particles sedimented on carbon-copper grids using a JEM 2100 microscope (JEOL, Tokyo, Japan) operating at 200 kV. Both the secondary electrons and microdiffraction regimes were employed.

Magnetic properties were measured at RT using a vibrating magnetometer (VSM, Cryogenics Ltd. London W3 7QE, UK) in a magnetic field up to 18 kOe. The samples were placed in gelatine capsule after mixing with GE varnish.

X-band ferromagnetic resonance (FMR) measurements were carried out on an ELEXSYS 500 spectrometer (Bruker, Karlsruhe, Germany) with a maximum available microwave power of 200 mW. It was equipped with a super-high-Q resonator ER-4123-SHQ. The spectra were recorded at room temperature using typical modulation amplitude of 1 Oe at a frequency of 100 kHz. The magnetic field (up to 10 kOe) was calibrated by a NMR probe and the frequency inside the cavity was determined with an integrated MW-frequency counter. Mirowave field and external magnetic field were perpendicular to each other in all cases under consideration. For microwave studies, NPs were mixed with a small amount of diluted GE7031 varnish. For the sake of comparison with our previous results on microwave behaviour of magnetic NPs [1,4,9] the measured derivative of the adsorbed power with respect to applied magnetic field (d*P*/d*H*) was integrated therefore *P*(H) spectra are the subject of the discussion in the present work.

## 4. Conclusions

Pure Fe_3_O_4_ and doped magnetite Zn_x_Fe_3−x_O_4_ magnetic nanoparticles were prepared by co-precipitation method and characterized by XRD, SEM, TEM and VSM techniques. Special attention was paid to microwave properties evaluation. Only one FMR resonance line was observed (*f* = 9.5 GHz) indicating that the materials are magnetically uniform. In all cases the width was large because of distributions of orientations, stresses and shapes. The deviation from the resonance field corresponding to spheres (~3.27 kOe) can be explained by taking into account dipolar forces, magnetoelastic contributions and magnetocrystalline anisotropy. The shape of the series B MNPs are close to being spherical. All samples show sizable zero field absorption. For A type samples the grain size decreases with an increase of the Zn content but zero field absorption and the grain size do not correlate. High zero-field absorption of A-I ferrite can be useful for biomedical applications as biocompatible iron oxide. For B type MNPs the grain size and zero field absorption show clear correlation. In both A and B series the highest zero-field absorption corresponded to 0.2 zinc concentration. Although, more structural and microwave studies are necessary (including the case of very diluted samples) in order to understand the details of the observed effects, it is clear that the microwave techniques are a powerful tool to characterize complex nanomaterials.
